# Attitude Towards Mental Illness among Medical Students and Interns of A Medical College

**DOI:** 10.31729/jnma.3716

**Published:** 2018-10-31

**Authors:** Astha Prasai, Shubash Chandra Sharma, Rika Rijal, Shreeyanta K.C.

**Affiliations:** 1 Kathmandu Medical College and Teaching Hospital, Sinamangal, Kathmandu, Nepal

**Keywords:** *attitude*, *interns*, *medical students*, *mental illness*

## Abstract

**Introduction:**

Mental health and physical health are interconnected. Attitude of medical professionals towards mentally ill affects the quality of care the patient receives. Today's medical students are tomorrow's medical practitioner. We conducted a study in a teaching hospital to assess the attitude towards mental illness among medical students and interns.

**Methods:**

A descriptive cross sectional study was performed in 265 students in Kathmandu Medical College in August, 2017. Opinion about Mental Illness (OMI) questionnaire developed by Cohen and Struening was used.

**Results:**

Of the total participants, 132 (49.8%) were male and 133 (50.2%) were female. Medical students were more authoritarian, more benevolent and believed that mental illness is like any other medical illness. They showed neutral attitude in terms of social restriction of mentally ill and interpersonal relationship as a cause of mental illness.

**Conclusions:**

Positive attitude was seen only in terms of benevolence and mental hygiene ideology. Effective teaching and training programs is necessary to bring in positive attitude change towards mentally ill among medical students.

## INTRODUCTION

Mental health is an inseparable part of overall health and well-being. Worldwide, 450 million people are suffering from some sort of mental illnesses.^1^ In Nepal, approximately 20–30% of adult patients visiting Primary Health Care services with somatic symptoms show psychiatric morbidity.^2^

Many mentally ill people remain undiagnosed or underdiagnosed because they hesitate to seek medical help due to the existing stigma and discrimination.^3^ Stigma exists even in the health care system.^4, 5^ This stigma affects patient care. A study among the medical students and interns in Kathmandu University School of Medical Sciences showed that the medical students and interns had neutral attitude towards people with mental illness.^6^

This study aims to assess the attitude of medical students and interns towards mentally ill which can help to evaluate the need for bringing in effective teaching and training programs to bring in positive attitude change.

## METHODS

This descriptive cross-sectional study was carried out in Kathmandu Medical College and Teaching Hospital in August, 2017. Ethical approval was obtained from the IRC of Kathmandu Medical College and Teaching Hospital (Ref: 02082017). Our study was conducted among MBBS students from first year to interns. Those unavailable at the time of study due to leave were not included in the study. All of them who gave consent were included in the study. Sample size calculation was done as follows:


$$\text{Sample size}({\text{n}}_{\text{o}})=\frac{{\text{Z}}^{2}\text{p(1-P)}}{{\text{e}}^{2}}$$

Where, Z= Z valve (1.96 with 95 % confidence level) P= Response distribution, similar research has not been performed in our setting, so response distribution is considered to be 50%

e= Margin of error, it is 5% (0.05)


$$\text{Thus, sample size}=\frac{1.96^{2}0.5(1-0.5)}{0.05^{5}}=384$$

$$\text{Now, True sample size =}\frac{{\text{n}}_{\text{o}}}{1+\frac{{\text{n}}_{0}-1}{\text{N}}}$$

here, n_o_ = sample size

N= total population, which is 851 as obtained from administrative records


$$\text{Thus, true sample size =}\frac{384}{1+\frac{384-1}{851}}=265$$

Total of 265 students were included. Simple random sampling technique was used to select the study population.

Data was collected by using Opinion about Mental Illness (OMI) questionnaire and a self designed socio-demographic questionnaire. It is a valid questionnaire devised by Cohen and Streuning. OMI consists of 51 items each scored on a six point likert scale ranging from 1 (strongly agree) to 6 (strongly disagree).

Social desirability bias was possible in this study, occurrence of which was eliminated by explaining the participants regarding the purpose of the study and that their responses will be kept confidential.

Descriptive statistics was used for analysis using SPSS version 20.

## RESULTS

Of the total 265 participants, 132 (49.8%) were males and 133 (50.2%) were females. Mean age of the participants was 21.67±1.85 years. One hundred and six (40%) of the respondents were from basic science and 159 (60%) were from clinical science. Most of them were Hindu 251 (94.7%) followed by Buddhist 11 (4.2%) and Muslim 3 (1.1%). When asked if they had sought any help for mental illness, 27 (10.2%) said they did.

**Table 1.** OMI score in medical students and interns.

Factor	Mean	Standard deviation	Range
Authoritarianism	33.17	±6.13	16–52
Benevolence	44.92	±6.63	26–60
Mental hygiene ideology	30.67	±4.00	20–42
Social restrictiveness	25.54	±5.08	13–40
Interpersonal etiology	19.76	±4.40	8–29

In terms of authoritarianism, highest possible score was 56 and lowest possible score was 1. Students had high score (33.17±6.13). In terms of benevolence, the score was high (44.92±6.63). Possible score ranged from -4 to 66. In terms of mental hygiene ideology, possible score was between 1 and 46. Students scored high (30.67±4.00). In terms of social restrictiveness, students had a neutral score (25.54±5.08). Highest possible score was 51 and lowest possible score was 1.

In terms of interpersonal etiology, students had neutral score (19.76±4.40). Possible score ranged from 1 to 36.

**Table 2.** OMI score (Mean ± Standard deviation) in medical students and interns by gender.

Factor	Male	Female
Authoritarianism	33.31±6.77	33.04±5.45
Benevolence	44.78±7.06	45.06±6.21
Mental hygiene ideology	30.24±3.90	31.09±4.07
Social restrictiveness	26.02±5.15	25.05±4.98
Interpersonal etiology	19.94±4.50	19.58±4.25

Male had higher score than female in terms of authoritarianism, social restrictiveness and interpersonal etiology.

**Table 3.** OMI score (Mean ± Standard Deviation) in medical students and interns by year of study.

Factor	First year	Second year	Third year	Final year	Intern
Authoritarianism	36.45±5.64	34.96±4.81	31.98±5.57	31.28±6.88	31.19±5.85
Benevolence	43.02±6.43	45.45±5.68	44.58±8.06	45.51±6.54	46.04±6.03
Mental hygiene ideology	31.43±4.43	31.09±3.93	30.81±4.03	29.51±4.00	30.49±3.40
Social restrictiveness	25.23±4.71	25.94±4.68	25.74±6.10	25.42±5.41	25.36±4.42
Interpersonal etiology	21.13±4.30	19.32±4.12	19.60±4.01	18.91±4.91	19.83±4.29

In terms of authoritarianism first year had higher score. In terms of benevolence interns had higher score than others. First year students had higher score in terms of mental hygiene ideology and interpersonal etiology. Scores on Social restrictiveness is almost similar.

Going through the individual items, 114 (43%) somewhat agreed that nervous breakdowns usually result when people work too hard. Only 93 (35%) participants agreed that mental illness is an illness like any other. Nearly half (47%) agreed that most people in mental hospital are not dangerous. Of the total, 88 (33%) believed that if the parents love their children more, there would be less mental illness. More than half (60.4%) strongly agreed and two of them (0.8%) strongly disagreed that even though patients in mental hospital behave in funny ways, it is wrong to laugh about them. About half (52%) agreed that many mental patients are capable of skilled labour even though in some ways they are very disturbed mentally. When asked if mental illness is caused by the separation or divorce of their parents during childhood, 116 (43%) strongly agreed to the statement. One hundred and nine participants (41%) disagreed to the statement that to become a patient in a mental hospital is to become a failure in life. One hundred and sixteen (43.8%) participants agreed that people who are unable to work because of mental illness should receive money for living expenses. More than half (52.8%) strongly agreed that our mental hospitals should be organized in a way that makes the patient feels as much as possible like he is living at home.

## DISCUSSION

People with mental illness experience stigma and discrimination not only in the general public but also in the health care system. People who stigmatize mentally ill people have a tendency to maintain a distance with them.^7^ Negative attitude towards mentally ill patient among medical personnel greatly affects patient care.^5^ At present, four out of five people with severe mental illness in Low and Middle Income Countries (LMIC) like Nepal receive no effective treatment.^8^

Many studies have been conducted in different parts of the world assessing the attitude of health personnel towards mental illness using different scales.^9, 10, 11^ In a study among forty five medical students in Tribhuvan University Teaching Hospital, Attitude Towards Mental Illness (AMI) score was found to be 66.93 indicating an overall positive attitude.^12^ In a study conducted among 106 nursing students in Mental hospital, Lagankhel Nepal using a different questionnaire, there was overall adequate knowledge and by large a positive attitude towards mentally ill people among the nursing students.^13^ In a study conducted by Risal et.al in Kathmandu University of Medical Sciences, Dhulikhel Nepal using ATP-30 and AMI questionnaire, most of the students showed neutral scoring towards all scoring items.^6^ A study conducted in Gujarat in a medical college showed that the undergraduate students have neutral attitude towards mental illness.^14^

In our study, we assessed the attitude of medical students towards mental illness in terms of five different factors using OMI questionnaire. In Factor A Authoritarianism, participants scored high reflecting a negative attitude in terms of this factor. First year scored higher compared to others. This indicates that the students see psychiatric patients as inferior and believe that they need strict handling.^15^ Similarly in a study using similar questionnaire conducted in a medical college in South India, improvement in stigmatization was seen after psychiatry posting.^16^ In Factor B Benevolence, participants had high scores. Interns had high score than other groups. High scores reflect a kindly paternalistic attitude toward psychiatric patients.^15^ Similar results were obtained in a study conducted in a tertiary hospital in North India by Gulati et.al using the same scale.^17^ In Factor C Mental hygiene ideology, participants had high scores which shows positive attitude in terms of this factor. This indicated the opinion that mental illness is similar to other illnesses and it should be treated adequately by specialists.^15^ First year students scored more than others in terms of Factor C. In contrast, interns considered mental illness to be like any other illness in a study conducted by Gulati et.al.^17^ In Factor D Social Restrictiveness, students had neutral attitude. High scores here would have indicated that the participant would like to see psychiatric patients restricted for protection of self and the society.^15^ Scores were similar in all the groups. In a study conducted in Pondicherri, India by Sujaritha et.al, more than half of the doctors had positive attitude towards social restrictiveness among mentally ill.^18^ In Factor E Interpersonal etiology, students had neutral score. First year students had high scores than others. High scorers would believe that the real cause of a mental illness is problematic interpersonal relations rather than a biochemical or genetic cause.^15^ In a study conducted among medical undergraduates in Karnataka, when asked about the etiology of psychiatric disorders, majority of the students attributed them to genetic reasons (59.9%) and imbalance of neurotransmitters (60.6%).^19^ In a study conducted in mental hospital Lagankhel, Nepal among 106 nursing students, majority had showed their knowledge about cause of mental illness as genetic (78.6%) and biochemical disturbances (97.2%).^13^

In this study, though the students showed positive attitude in terms of benevolence and mental hygiene ideology, they still have negative attitude in terms of authoritarianism and neutral attitude in terms of social restrictiveness and interpersonal etiology. This signifies either our teaching and training programme is not effective or the students are less concerned about mental health.

This study was performed in only one medical institution. So, it may not be representative of the entire MBBS students population of Nepal. Only MBBS students were considered in the study though it would be better if assessment of attitude among other faculties was also done. Comparison of attitude before and after clinical exposure was not done.

## CONCLUSIONS

To conclude, this study showed that MBBS students of Kathmandu Medical College have more authoritarian attitude, are more benevolent and view mental illness like any other illness. They showed neutral attitude with respect to social restrictiveness and interpersonal etiology. Students still have some misconceptions. This can directly affect the quality of patient care they will deliver in future. Interactive and effective teaching and training programmes since the basic science years is a must to further improve the attitude towards mentally ill.

## Conflict of Interest

**None.**
